# Elective Reconstruction of Perioral and Alar Defects Using a Subcutaneous Island Pedicle Flap: A Case Report

**DOI:** 10.7759/cureus.110785

**Published:** 2026-06-13

**Authors:** Mario Shuchleib Cukiert, Guillermo Roa Alvarez, Sergio Dib Fajer, Sergio Enrique Leal Osuna

**Affiliations:** 1 Department of Dermatology, Hospital General "Dr. Manuel Gea González", Mexico City, MEX; 2 School of Medicine and Health Sciences, Tecnológico de Monterrey, Mexico City, MEX; 3 Department of Dermatology, Skin and Hair Clinic, Mexico City, MEX; 4 Department of Dermatology, Hospital Español de México, Mexico City, MEX

**Keywords:** basal cell carcinoma, dermatologic surgery, island pedicle flap, nasal ala reconstruction, subcutaneous island flap, upper lip reconstruction

## Abstract

Subcutaneous island pedicle flaps are versatile reconstructive options in dermatologic surgery, particularly in cosmetically and functionally demanding facial regions. Their robust vascularity, preservation of adjacent aesthetic units, and ability to recruit tissue with similar color, texture, and thickness make them especially useful for reconstruction of defects involving the perioral region and nasal ala.

We report the case of an elderly woman with synchronous facial basal cell carcinomas involving the right upper cutaneous lip and the right alar groove. Following complete surgical excision, reconstruction was performed using a unilateral subcutaneous island pedicle flap (island flap) designed along the nasolabial fold. The flap provided adequate mobility to reconstruct both defects while maintaining the natural contour of the upper lip, alar-facial sulcus, and nasolabial fold. Postoperative evolution was favorable, with excellent vascular viability, preservation of oral competence, and satisfactory cosmetic integration at one month.

This case highlights the utility of the island pedicle flap as a single-stage reconstructive option for adjacent perioral and nasal defects, emphasizing principles of aesthetic subunit reconstruction and tissue conservation.

## Introduction

Reconstruction of facial defects after cutaneous oncologic surgery requires restoration of both function and aesthetics. Defects involving the upper cutaneous lip and nasal ala are particularly challenging because even minor distortions may alter oral competence, facial symmetry, nostril architecture, or the nasolabial fold [1-3].

The subcutaneous island pedicle flap, also known as the island flap, represents one of the fundamental reconstructive techniques in dermatologic surgery. The flap consists of an island of skin detached circumferentially while maintaining a central subcutaneous vascular pedicle that provides mobility and vascular supply [2,3]. Because the flap advances and rotates on a preserved pedicle, it offers excellent tissue match and allows scars to be concealed within natural facial creases [1,4].

Several authors have demonstrated the versatility of island pedicle flaps for reconstruction of defects involving the nose, cheeks, and lips, particularly after Mohs micrographic surgery [2-5]. Particularly in the perioral region, reconstruction is governed by the principle of aesthetic subunits, whereby preservation of the nasolabial fold, vermilion border, philtral architecture, and alar-facial groove is essential to achieve optimal cosmetic outcomes. Consequently, flap selection is often determined more by defect location than by defect size alone [1,5]. In the upper lip, the technique preserves the nasolabial fold, philtral symmetry, and vermilion border while minimizing distortion of adjacent structures [1,5]. Furthermore, island flaps can be adapted to repair complex defects involving neighboring aesthetic units, including the nasal ala and melolabial region [1,4].

We present a case illustrating the successful use of a subcutaneous island pedicle flap for simultaneous reconstruction of defects involving the right upper cutaneous lip and the alar groove following excision of two basal cell carcinomas. Although island pedicle flaps are widely used in facial reconstruction, reports focusing on their application for simultaneous defects involving adjacent perioral and nasal aesthetic subunits remain limited. This case illustrates how reconstructive planning based on aesthetic-unit preservation may influence flap selection and optimize cosmetic outcomes.

## Case presentation

A woman in her seventh decade of life presented for evaluation of two slowly enlarging lesions located on the right side of the face. Physical examination revealed a pearly papule with central ulceration involving the right alar groove and a second erythematous papule located on the right upper cutaneous lip adjacent to the melolabial fold.

Dermoscopic evaluation of both lesions demonstrated findings suggestive of basal cell carcinoma, including structureless pink areas and focal brown-gray pigmentation. No evidence of regional lymphadenopathy was identified.

**Figure 1:**
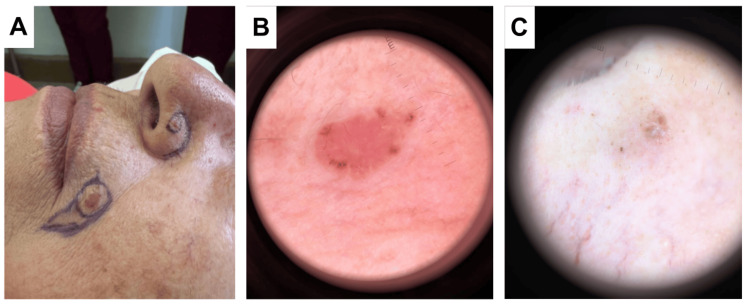
Preoperative clinical and dermoscopic evaluation (A) Clinical photograph showing two biopsy-proven basal cell carcinomas located on the right upper cutaneous lip and right alar groove. Surgical margins and flap design are outlined preoperatively. (B) Dermoscopic image of the upper lip lesion demonstrating a structureless pink background with focal brown-gray pigmentation. (C) Dermoscopic image of the alar lesion showing a pink structureless area with focal pigmented globules, findings consistent with basal cell carcinoma.

Preoperative planning included delineation of surgical margins and design of a reconstructive flap along the nasolabial fold (Figure 1). Following complete excision with histologically tumor-free margins, adjacent defects involving the right alar groove and upper cutaneous lip were created.

Given the close proximity of the defects and the need to preserve the natural alar-facial sulcus, upper lip contour, and nasolabial fold, reconstruction was performed using a unilateral subcutaneous island pedicle flap. The flap was designed within the melolabial region, with its vascular pedicle maintained in the subcutaneous plane. After adequate undermining, the flap was advanced and rotated medially into the defects. The donor site was subsequently closed primarily in a V-Y configuration.

**Figure 2:**
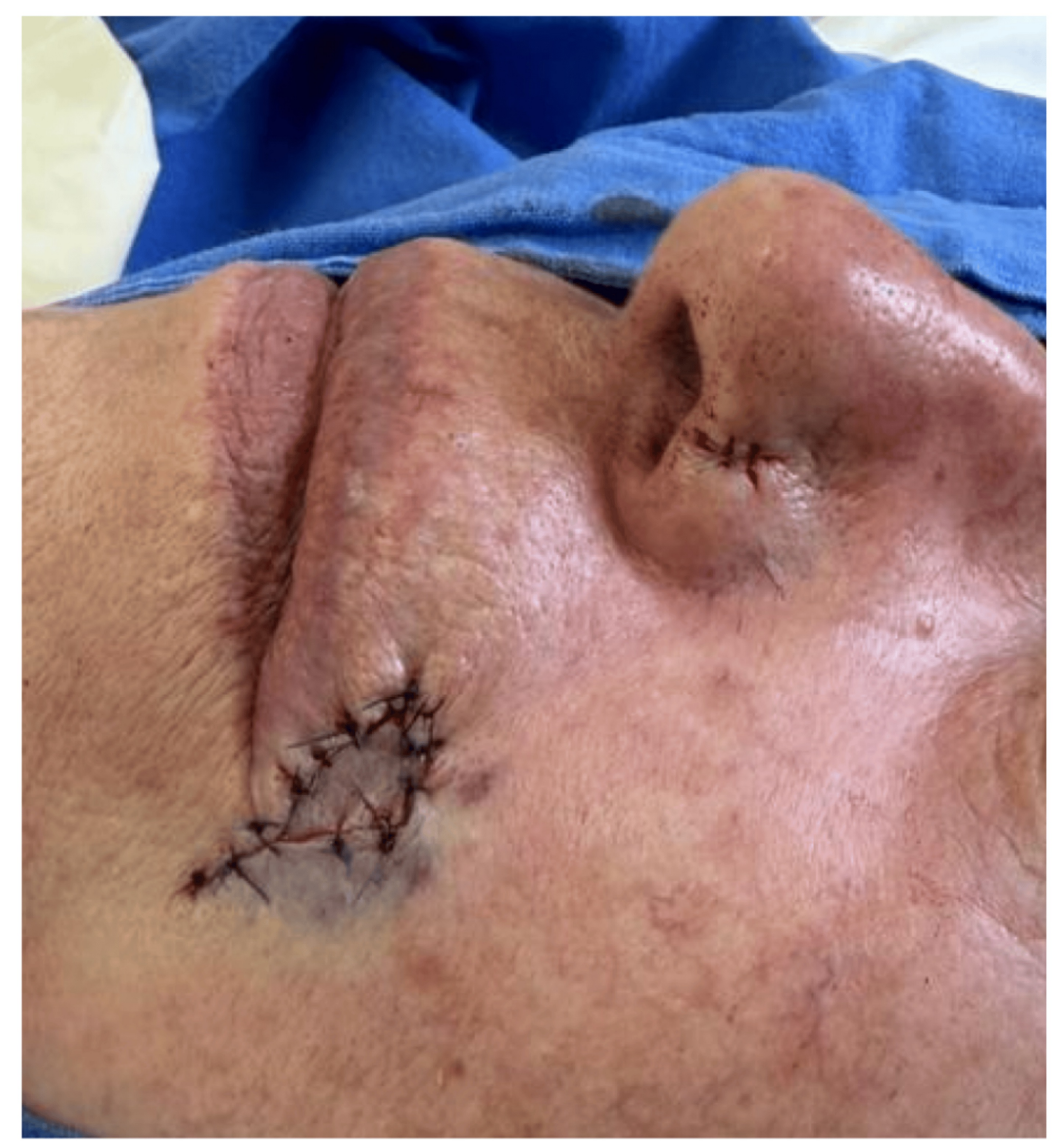
Immediate postoperative appearance following reconstruction with a subcutaneous island pedicle flap The flap was advanced from the melolabial region into the surgical defects. Adequate tissue perfusion and preservation of the alar-facial sulcus and upper lip contour are observed.

Immediate postoperative examination demonstrated excellent flap perfusion without evidence of vascular compromise (Figure 2).

**Figure 3:**
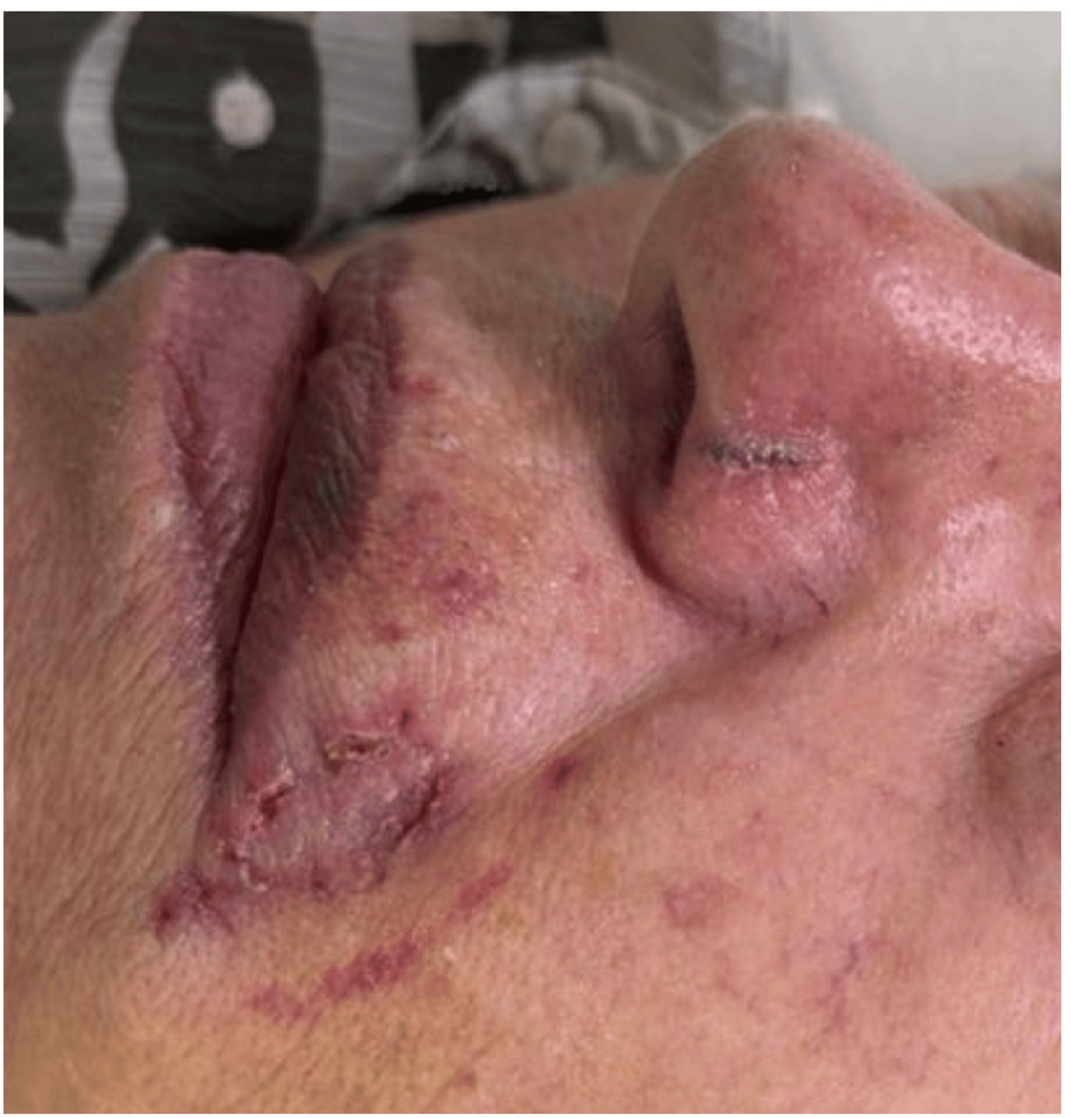
One-week postoperative follow-up The flap remains viable with satisfactory wound healing. Mild postoperative edema and ecchymosis are present without evidence of infection, necrosis, hematoma, or wound dehiscence.

At one-week follow-up, the flap remained viable with appropriate wound healing and no evidence of infection, necrosis, hematoma, or dehiscence. Mild postoperative edema and ecchymosis were observed, as expected during the early healing phase (Figure 3).

**Figure 4:**
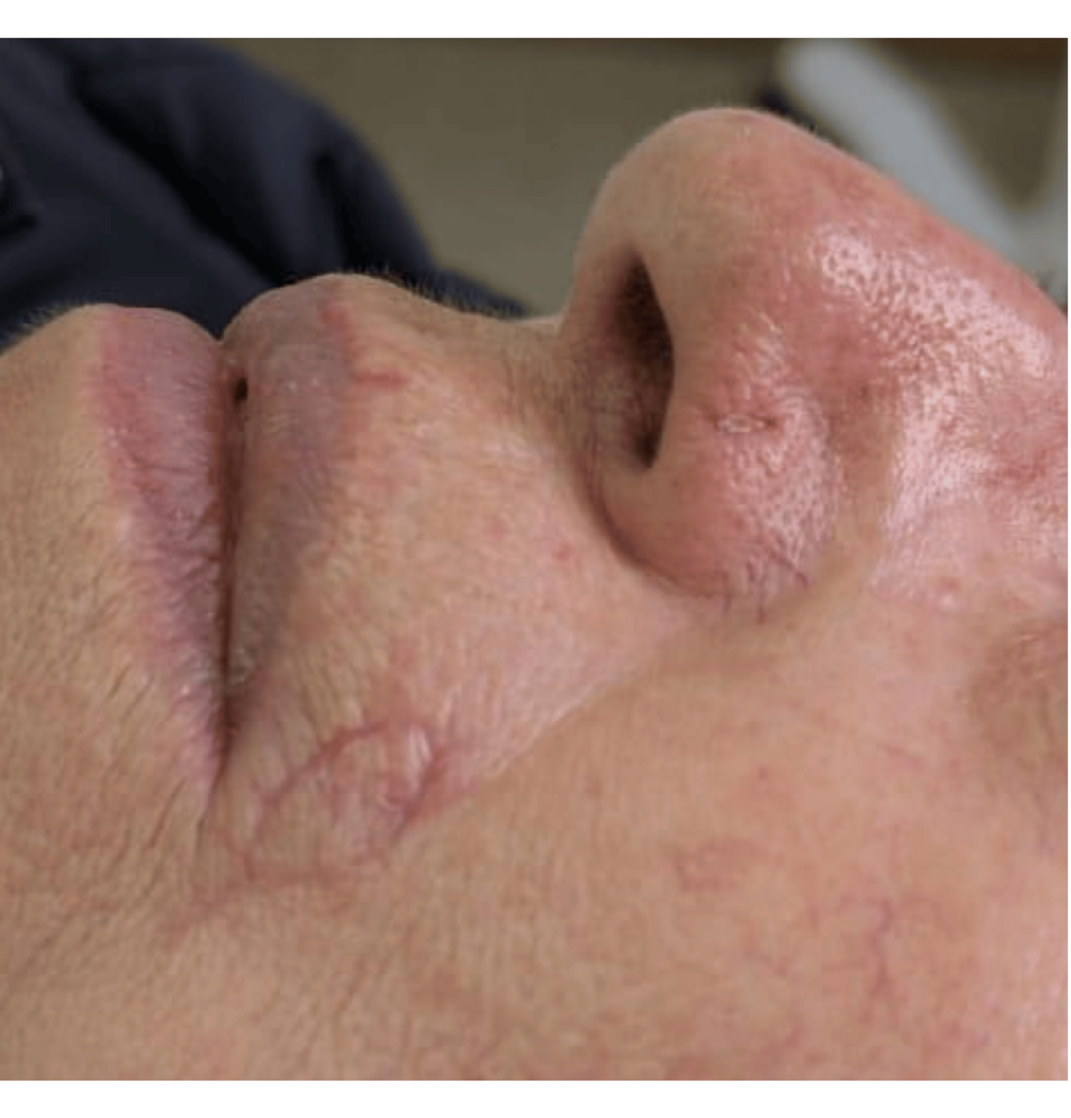
One-month postoperative follow-up Excellent cosmetic outcome with preservation of the nasolabial fold, upper lip contour, and alar architecture. Surgical scars are well concealed within natural facial creases.

At one month, reconstruction showed excellent integration with adjacent tissues. The alar groove and upper lip contour were preserved, scars were well concealed within the nasolabial fold and natural skin tension lines, and no trapdoor deformity, nostril distortion, or functional impairment was evident (Figure 4).

## Discussion

The selection of an optimal reconstructive technique after facial oncologic surgery requires careful consideration of both the defect characteristics and the aesthetic subunit involved. In facial reconstruction, the goal extends beyond simple wound closure and includes preservation of contour, symmetry, function, and the natural transition between adjacent cosmetic units [1,2].

The present case posed a particular reconstructive challenge because the two basal cell carcinomas were located within the right melolabial region, involving both the upper cutaneous lip and the alar-facial groove. These locations represent highly visible aesthetic subunits where even minimal distortion may compromise facial symmetry. Furthermore, the close proximity of the lesions created a combined surgical defect that required restoration of the upper lip contour while simultaneously preserving the alar-facial sulcus. 

Among the available reconstructive options, the subcutaneous island pedicle flap offered several advantages. This flap consists of a completely circumscribed skin island supplied by a centrally based subcutaneous vascular pedicle, allowing significant tissue mobility while maintaining reliable vascularization [2,3]. Unlike random advancement flaps, island flaps can recruit adjacent tissue with minimal tension and excellent blood supply, making them particularly useful in cosmetically sensitive facial regions. Although this technique is well established in facial reconstruction, its application in defects involving adjacent perioral and nasal aesthetic subunits provides an opportunity to discuss the reconstructive principles that guide flap selection.

Although frequently confused with a V-Y advancement flap, the island pedicle flap differs because the skin island is completely released circumferentially and remains attached only through a subcutaneous vascular pedicle. This configuration provides greater mobility, reduced tension, and the ability to combine advancement with rotational movement. In the present case, these characteristics facilitated preservation of the alar-facial groove and upper lip contour while allowing recruitment of adjacent tissue with minimal distortion.

One of the principal advantages of the island pedicle flap is its ability to provide tissue that matches the recipient site in color, thickness, texture, and sebaceous quality. This characteristic is particularly important in the perioral and nasal regions, where even subtle differences in skin quality may become conspicuous. In contrast, full-thickness skin grafts frequently produce contour irregularities, patch-like pigmentation differences, and secondary contraction, resulting in less satisfactory cosmetic outcomes [1,5,6].

The anatomical location of the defect was arguably the most important factor influencing flap selection in this case. The upper lip possesses several critical landmarks, including the vermilion border, philtral columns, nasolabial fold, and oral commissure. Similarly, the nasal ala contributes substantially to facial symmetry through its contour and relationship with the alar-facial groove. Reconstruction in this region must preserve these landmarks while avoiding distortion of the nostril aperture or upper lip architecture [1,4].

The island pedicle flap is uniquely suited for these situations because it recruits tissue from the melolabial fold, allowing scars to be concealed within natural facial creases. Tomás-Velázquez and Redondo emphasized that successful upper lip reconstruction depends largely on respecting aesthetic subunits and placing incisions along natural skin folds whenever possible [1]. The authors demonstrated that island flaps preserve nasolabial symmetry, maintain philtral architecture, and minimize visible scarring when properly designed.

Another significant advantage is the preservation of facial movement and oral competence. Because the orbicularis oris musculature remains intact and the flap is elevated primarily in the subcutaneous plane, functional impairment is minimal compared with larger rotational or transposition flaps [1,2]. In our patient, normal upper lip mobility and oral competence were preserved throughout follow-up.

The vascular reliability of the flap represents another major strength. The subcutaneous pedicle contains a robust vascular network that permits considerable advancement without compromising perfusion [2,3]. Consequently, flap necrosis is uncommon when an adequately thick pedicle is maintained. In the present case, complete flap survival was observed without ischemia, infection, hematoma, or wound dehiscence.

Despite its advantages, the island pedicle flap is not without limitations. Excessive advancement may produce distortion of adjacent structures or create tension vectors that alter the contour of the lip or nostril. Additionally, inadequate undermining can lead to the development of a trapdoor deformity caused by circumferential scar contraction and lymphatic obstruction [2,3]. Careful flap design, generous undermining, and preservation of a broad vascular pedicle are therefore essential technical considerations.

Alternative reconstructive options were considered. Primary closure would likely have generated unacceptable tension, resulting in distortion of the alar-facial groove and upper lip contour. Full-thickness skin grafting would have provided inferior color, texture, and thickness matching while carrying a greater risk of secondary contraction. V-Y advancement flaps, although effective for many perioral defects, generally provide less rotational freedom than island pedicle flaps and may be less suitable when simultaneous restoration of adjacent aesthetic subunits is required. Melolabial transposition flaps and cheek advancement flaps can provide adequate tissue volume but frequently generate longer scars and may alter the natural nasolabial contour [1,5]. For these reasons, an island pedicle flap was considered the most anatomically appropriate reconstructive option in this patient.

Although island pedicle flaps are commonly employed in facial reconstruction, reports often focus primarily on technical execution. The educational value of the present case lies in demonstrating the reconstructive decision-making process in a defect involving two adjacent aesthetic subunits. This case highlights how preservation of the alar-facial groove, nasolabial fold, and upper lip contour can influence flap selection and ultimately determine cosmetic outcomes. We believe this discussion may be valuable for dermatologic surgeons facing similar reconstructive challenges.

The postoperative outcome further illustrates the aesthetic benefits of this technique. At one month, the reconstructed region demonstrated preservation of the alar-facial groove, maintenance of upper lip symmetry, and inconspicuous scars hidden within natural facial creases. These findings are consistent with previous reports demonstrating favorable cosmetic and functional outcomes following island pedicle flap reconstruction of upper lip and adjacent nasal defects [1,4-6].

## Conclusions

The subcutaneous island pedicle flap is a highly effective reconstructive option for facial defects involving the upper cutaneous lip and nasal ala. Its excellent vascularity, versatility, and ability to recruit adjacent tissue with optimal color and texture match make it particularly valuable in dermatologic surgery. In this case, the flap provided restoration of both form and function while preserving the natural anatomy of the nasolabial fold and alar-facial sulcus. Dermatologic surgeons should consider the island pedicle flap among their primary reconstructive options when managing adjacent perioral and nasal defects following skin cancer excision.
